# Visualization and Analysis of 3D Microscopic Images

**DOI:** 10.1371/journal.pcbi.1002519

**Published:** 2012-06-14

**Authors:** Fuhui Long, Jianlong Zhou, Hanchuan Peng

**Affiliations:** Janelia Farm Research Campus, Howard Hughes Medical Institute, Ashburn, Virginia, United States of America; Whitehead Institute, United States of America

## Abstract

In a wide range of biological studies, it is highly desirable to visualize and analyze three-dimensional (3D) microscopic images. In this primer, we first introduce several major methods for visualizing typical 3D images and related multi-scale, multi-time-point, multi-color data sets. Then, we discuss three key categories of image analysis tasks, namely segmentation, registration, and annotation. We demonstrate how to pipeline these visualization and analysis modules using examples of profiling the single-cell gene-expression of *C. elegans* and constructing a map of stereotyped neurite tracts in a fruit fly brain.

## Introduction

**Figure 1:**
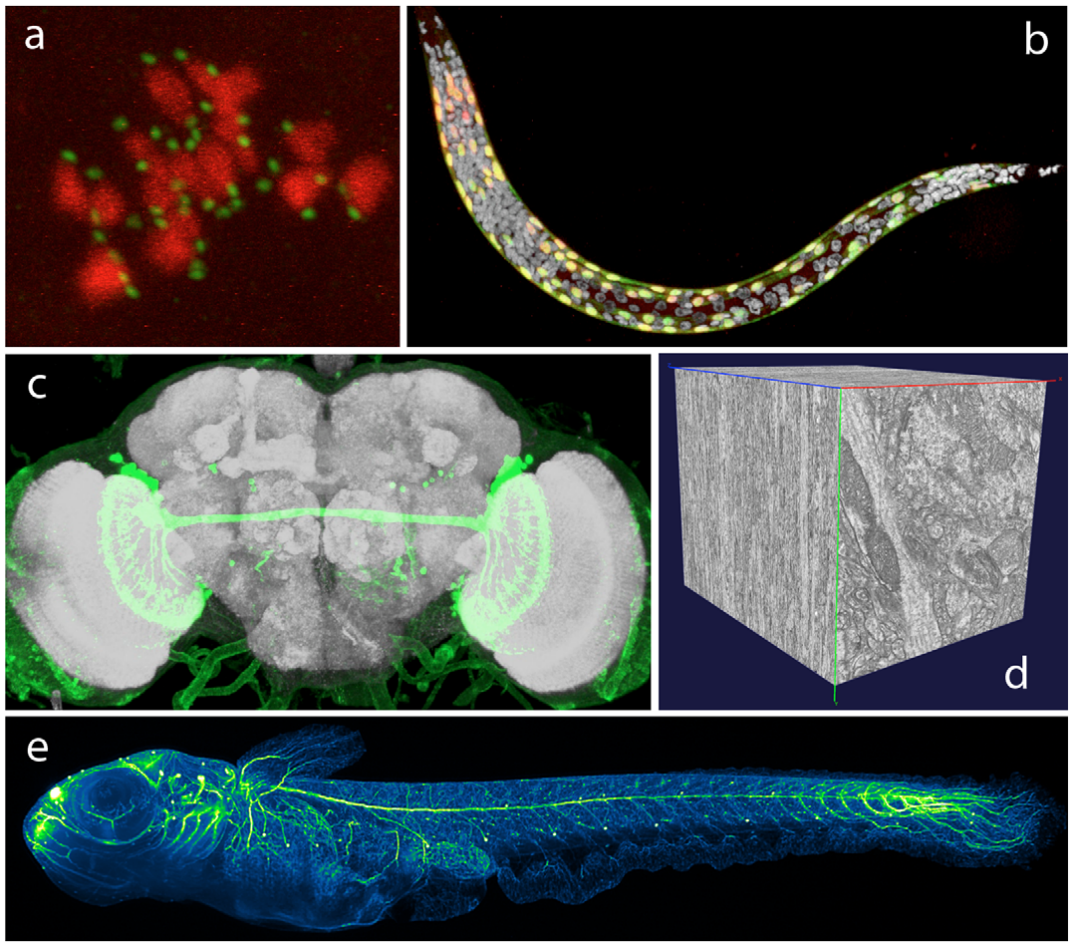
Examples of 3D microscopic images. (a) A confocal image of kinetochores (EGFP labeled) and chromosomes (histone-mCherry labeled) used in studying the first meiotic division in mouse oocytes [17]. (b) A confocal image of the first larval stage of *C. elegans* [18]. Gray: DAPI labeled nuclei; yellow: myo3:EGFP. (c) A confocal image of an adult fruit fly brain [19]. Gray: NC82 labeled neuropil; green: ato-GAL4 (courtesy of Julie Simpson). (d) A serial section electron microscopic image of mouse visual cortex [20]. (e) A digital scanned laser light sheet fluorescence microscopic image of a Medaka juvenile [21]. Green: acetylated tubulin immuno-staining of the developing brain and spinal cord.

Multidimensional microscopic image data sets (Figure 1) are widely used in modern biology studies, especially in screening various phenotypic data. Analyzing microscopic data is highly useful and fruitful, such as observing the dynamics of microtubule spindles during mitosis [1], profiling gene expression of cells [2]–[4], and reconstructing the three-dimensional (3D) morphology of neurons [5]–[7]. Image visualization also enables effective development of high-content high-throughput bioimage informatics techniques [8] to extract biologically meaningful knowledge from microscopic images. It is also critical for visualizing raw images and respectively processed results (in terms of surface objects).

In this primer, we briefly introduce the basic concepts and methods of 3D microscopic image visualization and analysis, which are the two core components for a number of bioimage informatics applications. We emphasize fluorescent microscopic images as examples, and occasionally also mention other types of image data in our discussion. On the other hand, the essential visualization and analysis methods introduced here can be applied to a wide range of data, including many of those not explicitly discussed. Due to the length limitations of this educational note, here we do not intend to comprehensively survey software tools or biological applications, which can be found in a few previous reviews [8]–[11].

## Visualization of 3D Microscopic Images

Visualizing 3D microscopic images helps better understand the data. It also helps determine appropriate analysis methods or parameters. In addition, visualizing analysis results on top of, or side-by-side with, the input image(s) is critical for checking the meaningfulness of an analysis and making necessary corrections (“proof-editing” [12]).

Two-dimensional (2D) cross-sectional display (Table 1) of a 3D image stack is still the most prevailing method for biologists to observe 3D data sets, probably due to its simplicity. ImageJ [13] (a newer variant bears the name Fiji), a popular tool to visualize and analyze microscopic images, uses mainly the z-section display to visualize 3D images, although various additional ImageJ modules or plugins were also developed to render 3D views. Tri-view display (Table 1) is a natural extension of the z-slice display of 3D data, displaying all XY, XZ, and ZY cross-sectional planes at the same time. Cutting through the volumetric data from an arbitrary angle and displaying the 2D image data on this cutting plane is also useful. These features have been incorporated in other scientific visualization software packages (e.g., Vaa3D (previously known as V3D [14]) or GoFigure [15]). Electronic microscopic (EM) images typically have a large cross-sectional size in the XY plane. It is particularly convenient to view EM images using 2D or tri-view display methods, such as in the ImageJ-based software TrakEM [16].

**Table 1:** Often-used visualization methods for multi-dimensional microscopic image data.

Visualization mode	Dimension	Often Applied to	Pros	Cons	Example Figure(s)
***Cross-sectional view(s)***	2D	FM/WM/EM	Fast	Not 3D	—
***Tri-view***	2D	FM/EM	Fast	Partial 3D	3a
***Maximum intensity projection (MIP)***	3D	FM	3D	Hardware-limited (HL)	1a, 1b, 1c, 1e, 2b
***Alpha-value blending***	3D	FM/EM	Surface-display effect	HL	1d, 2a
***Multi-channel/multi-color 3D (MC-3D)***	4D	FM	3D	Need color-blending (CB), HL	1a, 1b, 1c, 1e, 2b
***Multi-time-point MC 3D (MT-MC-3D)***	5D	FM	3D	CB, HL	2b
***Multi-scale MT-MC 3D (MS-MT-MC-3D)***	6D	FM	Hardware-friendly, 3D	Need 3D interaction of image content	2a
***3D surface-object and image rendering***	Heterogeneous 3D/4D/5D/6D	FM/EM	Allow proof-reading/editing	HL	2a, 4b

CB, color-blending; EM, electron microscopic images; FM, fluorescent microscopic images (often laser-scanning-microscopic images); HL, hardware-limited; WM, wide-field light microscopic images.

However, cross-sectional views are not able to visualize the 3D information of volumetric images. Visualizing the *complete* 3D information in a volumetric image requires seeing (a) all individual image voxels' (pixels) intensity, and (b) the 3D spatial adjacency information of all voxels. However, since normally a rendered image is a 2D projection to a computer screen and our retina, it is hard to meet both requirements at the same time. Tiling all image voxels on a single 2D plane will not appropriately display the 3D spatial adjacency information. On the other hand, in a 3D volumetric rendering, while the spatial adjacency relationship is retained, not all image voxels' intensity is visible, as voxels near the viewer will occlude far-away voxels. Therefore, selectively discarding the non-important voxel intensity information is the central trick used in 3D volumetric image visualization.

3D image visualization calls for depth-blended views from any angle. Maximal (or minimal) intensity projection (MIP or mIP) and alpha-value blended views (Table 1) are two main types of methods to display 3D data. MIP is mainly used to visualize high-intensity structures within volumetric data. This is the typical situation for most fluorescent microscopic (FM) images, e.g., GFP-labeled neuron structures. Usually, MIP contains no shading information; depth and occlusion information are not visible. Structures with higher intensity value lying behind a lower valued object appear to be in front of it. Thus, MIP may not accurately display the actual 3D relationships of structures. While alpha-blended views can display the depth information more meaningfully, the most common solution is to animate or interactively change the viewpoint while viewing using MIP (or even alpha-blended views). Therefore, a real-time 3D renderer for large datasets is highly desirable. This often needs both good hardware (i.e., high-throughput graphics card with large memory) and optimized software (e.g., to optimize the OpenGL-based graphics rendering). Vaa3D (http://vaa3d.org) meets this requirement and has been used in recent large-scale 3D image visualization applications, e.g., the Janelia Fly WorkStation that currently screens more than 50 terabytes of fruit fly brain images (private communication with the Janelia FlyLight project).

In many cases, each voxel in a 3D microscopic image could have multiple color components that correspond to various features of the biological entities (e.g., different fluorephores with different wavelengths in fluorescent imaging). Visualizing multi-channel (MC) 3D image stacks (thus four-dimension [4D], see Table 1) requires blending the data in different channels to the RGB space for rendering. When the number of channels, *N*, is not larger than 3, a simple mapping, e.g., channel 1 to Red or Magenta, channel 2 to Green, and channel 3 to Blue, is often used. When *N*>3, e.g., in the cases of dozens of co-localized antibody-probes, or thousands of 3D registered image stacks organized as different channels [19], a spreadsheet-based color-blending manager (e.g., the one provided in Vaa3D) will be critical for effective visualization.

**Figure 2:**
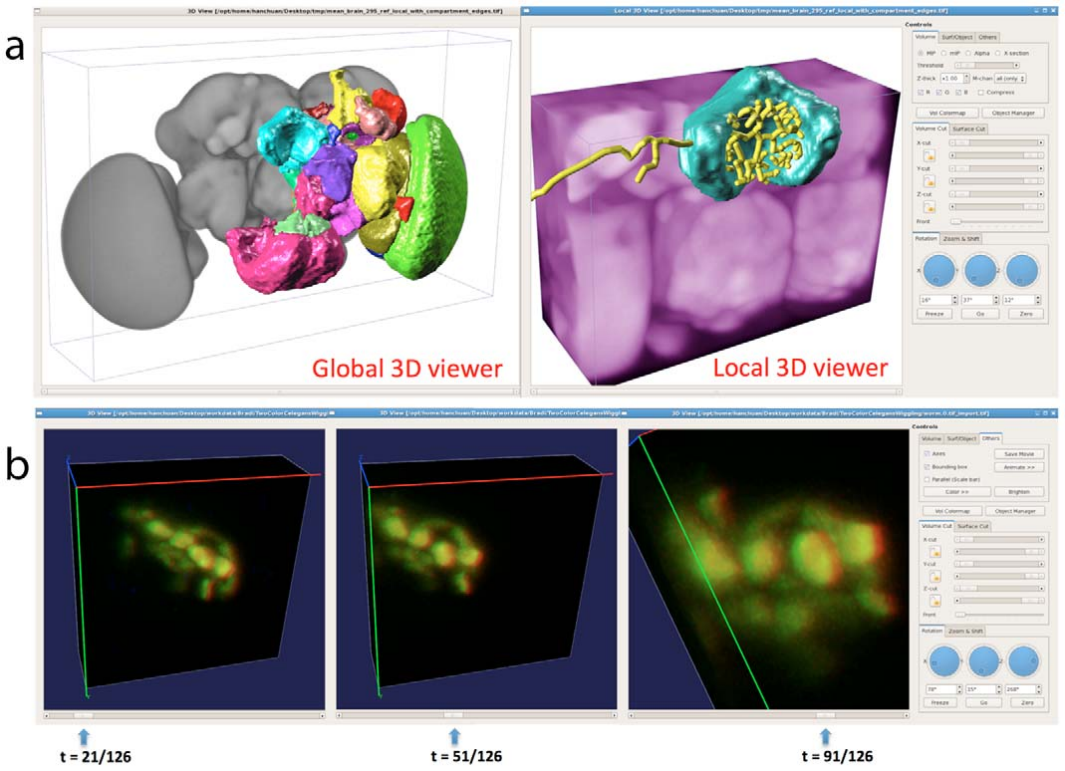
Vaa3D visualization of 4D and 5D microscopic images, as well as associated 3D surface objects, of different model animals. (a) The hierarchical (multi-scale) 3D visualization of a fluorescent confocal image of fruit fly (*Drosophila melanogaster*) brain using both global and local 3D viewers. In the global viewer, different brain compartments rendered using surface meshes (in different colors) are overlaid on top of the 3D volume of a fruit fly brain. When an image is very large, the global viewer can serve for navigation purpose. A user can quickly define any 3D local region of interest and display it in a local 3D viewer using full resolution. In this example, the brain voxels can be rendered in a different color from the global viewer, while the user can optionally display other surface objects, such as the single 3D-reconstructed neuron (yellow). (b) 5D visualization of a series of multi-color 3D image stacks of *C. elegans* (courtesy of Rex Kerr). Different 3D viewing angles can be adjusted in real-time in Vaa3D, with which the user can freely change the displayed time point (bottom).

Live imaging experiments produce multi-time-point (MT) multi-color 3D image series (thus five-dimension [5D], see Table 1). In addition, when an image is large (e.g., 20 Gbytes/image), it is usually impractical and also unnecessary to load all image voxels in the computer memory and graphics card to visualize. Thus, there is a need to visualize an image dataset at multiple scales. The MT-MC-3D data sets, and multi-scale (MS) rendering (thus six-dimensional visualization [6D], see Table 1), impose significant challenges to current visualization hardware and software, due to the limited bandwidth between hard drives, computer memory, and graphics card. When the entire image series could be loaded in computer memory, Vaa3D could be used to produce real-time 5D or 6D rendering (Figure 2). Yet, in general they are unsolved problems for terabyte-sized image data sets.

Surface-object rendering (Table 1) is a powerful way to visualize image analysis results (e.g., image segmentation) and provides ways for quantitative measurement or editing. Isosurface-based mesh-extraction and rendering has also been used in 3D biomedical image visualization. However, commonly used algorithms, e.g., marching cubes [22], are computationally expensive. In addition, isosurfaces can hardly capture the internal structures in a 3D image.

Interactive visualization techniques are important for microscopic image analysis. Through interactions, users can collect much more information of the multi-dimensional data than passively observing the 3D rendered data. Interacting with 3D rendered surface-objects is straightforward. It is more difficult to directly interact with 3D rendered volumetric data to define interesting 3D locations, 3D curves, and other objects. The concept of 3D-WYSIWYG (what you see is what you get) was recently proposed in the Vaa3D system to define an unambiguous 3D location (point) using one computer mouse click, or define a unique 3D curve using one mouse stroke on the 2D computer screen. This approach has been demonstrated to boost both the reconstruction speed and accuracy of 3D neuron morphology [12], [14]. In the long run, integrating these 3D interaction techniques in immersive visualization of very large data, possibly also equipped with other virtual reality techniques and a very large display wall, may demonstrate its power in detecting interesting patterns or associations in very large data sets.

In practice, 3D visualization of multi-dimensional image data may involve many other considerations. For instance, in both 3D tomographic EM imaging and laser scanning microscopy, anisotropy is an often seen property of the data. Software tools (e.g., Vaa3D) can reslice the data in the 3D rendering based on the relative pixel size in three dimensions, thus providing a more realistic display of the data. In Vaa3D, this auto-slicing function is combined with some image analysis functions (e.g., fibrous structure tracing) discussed below to generate various 3D reconstructions of the image objects. In addition, data filtering techniques (e.g., non-linear anisotropic diffusion, recursive median filtering, bilateral filtering, etc.) have been provided in many software tools (e.g., ImageJ). Integrating all these tools together could lead to more interesting insight in the data (see the last section on “pipelining”).

## Analysis of 3D Microscopic Images

The overarching goal of microscopic image analysis is to quantitatively measure “objects” in microscopic images, preferably in an automatic manner. Various labeled molecules (e.g., proteins or protein complexes), sub-cellular organelles, cells, or super-cellular objects (e.g., neuron populations or cell lineages) often need to be extracted, named, and compared with each other, before they can be measured. Most microscopic image analysis techniques can be categorized into three major classes, namely *segmentation, registration*, and *annotation*.

- Segmentation is the process of partitioning an image into multiple regions, so that voxels within each region share certain common features. Image segmentation is often used to locate objects and their boundaries (lines, curves, etc., e.g., [23]), as well as to perform qualitative and quantitative analysis in images [24], [25]. In microscopic image analysis, segmentation is typically used to locate, track, and classify bio-structures such as cells or nuclei [4], fibrous structures (e.g., axonal fibers [5], [7], microtubules [26]), and anatomical/functional tissue regions. Thresholding [27], watershed [28], [25], and deformable models [29] are the basis for the most commonly used segmentation techniques for microscopic images.
- Registration [30] is the process to map multiple or many images geometrically, via a linear or nonlinear transform, so that image objects or features can be compared directly in a “standard” space. Registration is particularly widely used in three types of microscopic image processing tasks: stitching of image tiles [31] (e.g., electron and light microscopic tiles), registration of multiple samples of the same biological entity [19], [32] (e.g., different images of the same neuron-population), fusion of multi-different views [33] of one object (e.g., tomography used in electron microscopy or selective plane illumination microscopy). Rigid or affine transforms are often used to register images globally. These linear transforms can be iteratively applied to images at different scales to achieve nonlinear registration. However, it is more common to use B-spline or thin-plate-spline to derive nonlinear smooth transforms [34], which are often used to register images locally.
- Annotation is the process to label/name images or image objects (e.g., cells) or assign their phenotypic properties with predefined terms. For example, controlled vocabularies of ontology have been assigned to images for annotating gene expression patterns (e.g., [35]–[37]). Another significant type of application is to recognize special objects of interest (e.g., cells) automatically [4], [38] and therefore to facilitate the quantitative measurement of biological entities (e.g., single-cell resolution gene expression).

## Pipelining 3D Visualization and Analysis Modules

**Figure 3:**
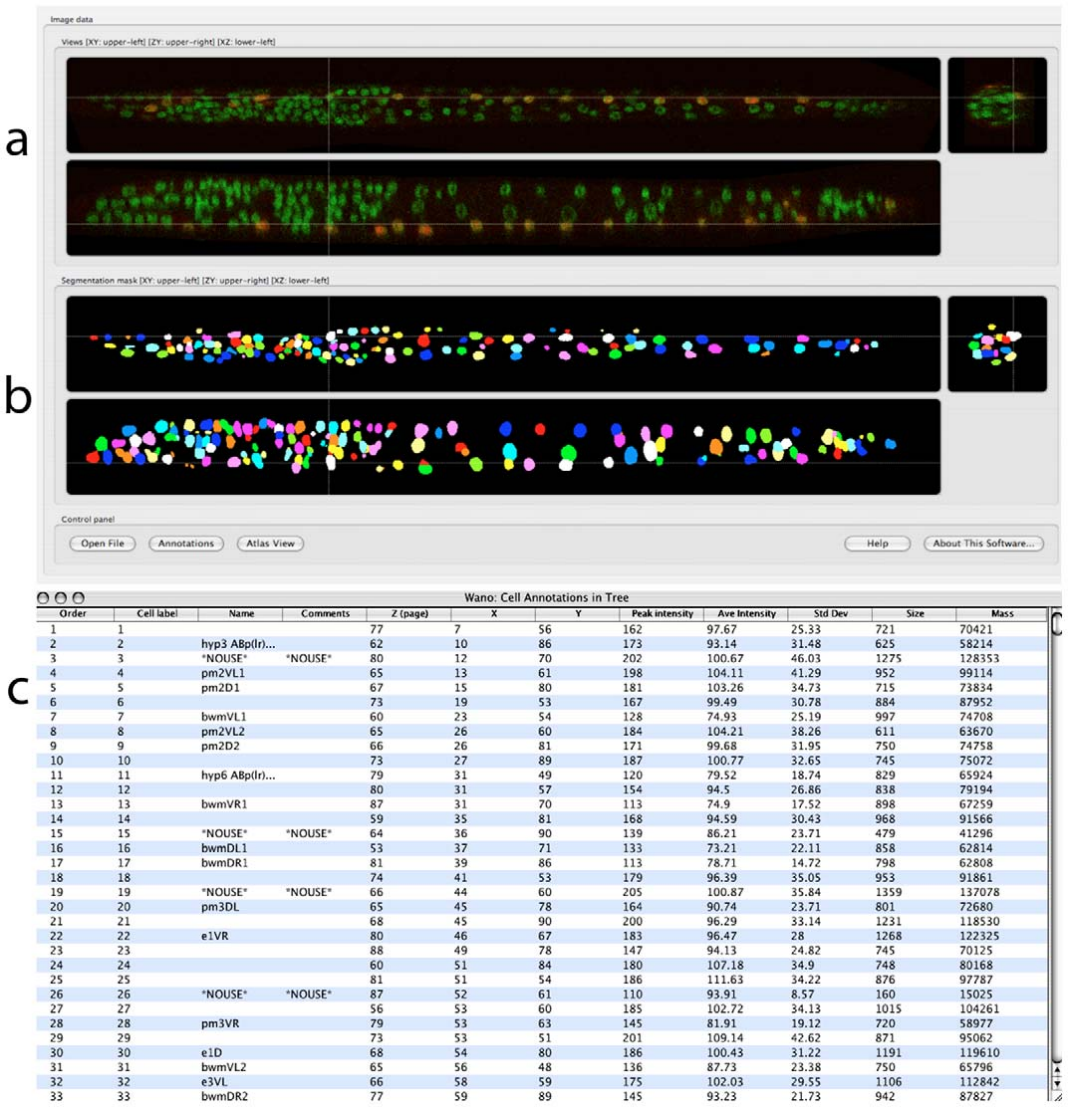
3D image visualization and analysis for measuring single-cell gene expression of *C. elegans*. (a) Tri-view display of a confocal image of *C. elegans* (L1 stage). Green: DAPI staining (pseudo-colored); red: myo3:GFP labeled muscle cells. (b) Tri-view display of the 3D watershed segmented nuclei of (a). The co-localized image objects are indicated by crosses (white). (c) A spreadsheet display of 3D measured gene expression of various cells. All sub-figures are produced using VANO [39], a 3D annotation tool.

In many biological applications, different image analysis techniques need to be used as a whole pipeline. For instance, for profiling the gene expression at the single nucleus resolution of *Caenorhabditis elegans* [4], laser scanning microscopic images of this animal are first straightened (Figure 3a) [40], which can be categorized as a registration step. Then, *C. elegans* cells that are stained using DAPI are segmented (Figure 3b) using an adaptive 3D watershed algorithm. Cells are then recognized (Figure 3) based on their relative location patterns in the 3D standardized space. Once the cell identities are determined, quantifying the gene expression is as simple as computing the normalized intensity within the nucleus region. The segmentation and recognition steps can also be unified using a recent approach of atlas-to-image deforming model [38].

**Figure 4:**
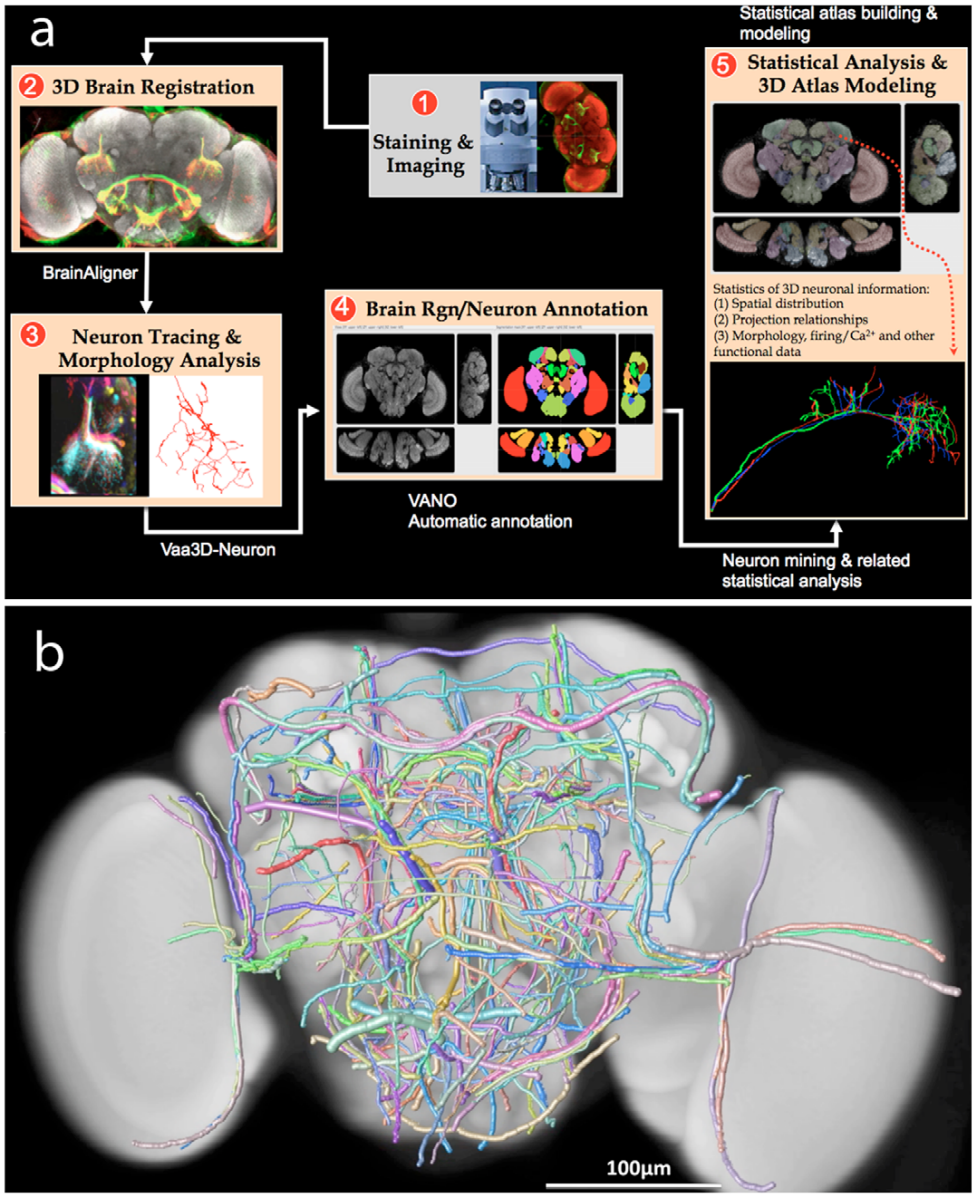
A pipeline of image analysis and data mining tools for building the neuronal atlases of fruit fly brains. (a) A flowchart of the key steps in building a fruit fly brain atlas. (b) A 3D digital atlas of 269 stereotyped neurite tracts reconstructed from GAL4-label fruit fly brains [19]. Pseudo colors are used to distinguish different tracts. The width of each tract equals its spatial divergence.

Pipelining image analysis modules and other more sophisticated data analysis/mining modules is a powerful way to generate quantitative biology. One such pipeline is shown in Figure 4a, which illustrates the main steps to construct the first 3D map of spatially invariant neurite tracts of a brain. Confocal images of adult fruit fly brains are first registered in 3D using the BrainAligner system [19] (Figure 4a, Step 2), so that different populations of neurons labeled using a number of GAL4 lines can be aligned and compared within the same 3D coordinate system. Then, neurite tracts are segmented and traced in 3D (Figure 4a, Step 3). The neurite tracts reconstructed from the same GAL4 line have a clear correspondence. They are then annotated (Figure 4a, Step 4). A neuron/neurite comparison and mining module is then used to determine the spatial divergence of the corresponding neurite tracts (Figure 4a, Step 5), followed by a final mapping to the standard space of the 3D fly brain atlas (Figure 4a, Step 5). With this approach, it is possible to measure hundreds of stereotyped neurite tracts in a fruit fly's brain (Figure 4b). The same pipeline can be used to study other brain wiring-maps of neurons.

## Conclusions

Visualization and analysis methods are critical for understanding and using 3D microscopic images for various cell biology, structural biology, neurosciences, and systems biology applications. These tools become indispensable for the ever-increasing need to screen tens of gigabytes to many terabytes of microscopic images. Pipelining these tools and other data analysis/mining methods is a new trend for producing interesting biology.
